# Research on the Pavement Performance of Fiber-Reinforced High Modulus Asphalt Concrete

**DOI:** 10.3390/polym18030365

**Published:** 2026-01-29

**Authors:** Gaixia Chen, Tuanjie Wang, Yuquan Yao

**Affiliations:** 1 School of Architectural Engineering, Zhengzhou University of Industrial Technology, Zhengzhou 451100, China; chengaixia@zzuit.edu.cn (G.C.); wangtuanjie@zzuit.edu.cn (T.W.); 2 School of Civil Engineering and Architecture, East China Jiaotong University, Nanchang 330013, China

**Keywords:** high modulus asphalt concrete, fiber, pavement performance, reinforcing materials

## Abstract

Under high temperature and heavy load conditions, asphalt pavements are prone to rutting and other distress, which severely affect the service life of the road. High modulus asphalt concrete has significant advantages in addressing rutting issues in asphalt pavements. However, its low-temperature performance is often poor, especially in regions with hot summers, cold winters, and large diurnal temperature variations, which limits the application of this technology. Based on this, the study introduces three types of fibers: basalt fiber, polyester fiber, and lignin fiber as reinforcing materials to improve the performance of high modulus asphalt concrete. The effects of these fibers on the pavement performance of high modulus asphalt concrete are systematically evaluated through rutting tests, low-temperature bending tests, immersion Marshall tests, freeze–thaw splitting tests, fatigue tests, and dynamic modulus tests. The test results show that as the fiber content increases, the effect of the fibers on the high-temperature, low-temperature, and fatigue performance of high modulus asphalt concrete initially improves and then decreases. The impact on water stability is not significant, while the dynamic modulus performance decreases. Fibers can significantly improve the low-temperature performance of the mixture. Among them, basalt fiber shows the greatest improvement in high-temperature and fatigue performance, while polyester fiber provides the best improvement in low-temperature performance. The improvement effect of lignin fiber is not as pronounced as that of the first two fibers. All types of fibers have an adverse effect on the dynamic modulus of the mixture. Taking all factors into consideration, the recommended fiber contents for basalt fiber, polyester fiber, and lignin fiber are 0.4%, 0.3%, and 0.3%, respectively, as these levels exhibited the best overall performance among the discrete dosages investigated in this study. Based on the experimental results, and within the selected dosage range, a performance evaluation system for fiber-reinforced high modulus asphalt concrete is established.

## 1. Introduction

With the global economic development and the continuous increase in traffic volume, roads of all classifications face higher structural load requirements and more demanding service conditions [1,2,3]. Asphalt pavements are widely used in high-grade highways due to their strong load-bearing capacity, smooth and safe driving, fast construction, and easy maintenance. However, traditional asphalt mixtures are prone to rutting, structural deformation, and early damage under long-term high temperatures and repetitive vehicle loading, leading to reduced road lifespan and increased maintenance costs [4,5]. Therefore, exploring new materials with higher modulus and better structural load-bearing capacity has become an important research direction in the field of road engineering [6].

High modulus asphalt concrete (HMAC) has become one of the effective solutions for addressing road rutting distress due to its excellent high-temperature deformation resistance [7,8]. High modulus asphalt concrete was first proposed by France. The core concept is to increase the high-temperature dynamic modulus and stiffness of the material, thereby reducing the strain generated on the pavement under high-temperature coupled vehicle loading, increasing the load distribution angle to disperse the stress, reducing surface deformation, and suppressing rutting distress [9,10]. Practical engineering has proven that high modulus asphalt concrete can effectively extend the service life of roads [11,12]. However, high modulus asphalt concrete has relatively poor flexibility, which often negatively affects the low-temperature crack resistance of asphalt pavements. When used in regions with large temperature differences and severe cold, it is prone to cracking and other low-temperature distresses, threatening driving safety and reducing the service life of the pavement, thus limiting its widespread application.

Fiber-reinforced asphalt concrete has been proven to be an effective method for addressing low-temperature damage in asphalt pavements. Wu et al. [13] conducted a comprehensive review of fiber-reinforced asphalt concrete technology, focusing on the use of fibers in asphalt mixtures. The review covers topics such as fiber types, dosage, incorporation methods, performance evaluation, enhancement mechanisms, and the application of various fibers in practical engineering projects. Kim Min-Jae [14] conducted experiments by incorporating various fibers into asphalt mixtures and found that the mixture with basalt fiber and polyester fiber of 6 mm in length exhibited the best performance. Ali Raza Khan et al. [15] studied the use of four types of fibers in the preparation of asphalt mixtures. The results indicated that the fibers significantly enhanced the durability of the asphalt mixtures. Sabouri et al. [16] used polyester fiber, glass fiber, and polypropylene fiber to address the mechanical performance deficiencies of cold recycled asphalt mixtures. They also modified the IDEAL-CT cracking index to better evaluate the crack resistance of asphalt mixtures. Shambilova et al. [17] investigated the preparation of PP-based macrofibers from recycled polypropylene (PP) and examined the effects of the antioxidant vitamin E on the rheological behavior of polypropylene melts and the mechanical properties of the fibers. The results indicate that the addition of a small amount of vitamin E can improve the processing stability and fiber spinnability of recycled polypropylene, providing important guidance for the preparation of high-performance macrofibers.

Different types of fibers, such as basalt fiber, polyester fiber, and lignin fiber, can significantly affect a series of mechanical properties of asphalt mixtures, including high-temperature rutting resistance, low-temperature crack resistance, dynamic modulus, and fatigue performance, by improving the internal distribution and stress-transfer characteristics at the aggregate–asphalt interface. Specifically, the study by Zhang et al. [18] systematically analyzed the effects of fiber characteristics (type, morphology, and content) on the performance of asphalt mixtures, emphasizing the importance of fiber morphology and its interaction with the asphalt matrix in enhancing performance. In addition, Wu et al. [19], through experimental comparisons of SMA-13 asphalt mixtures reinforced with different fibers, further confirmed the comprehensive improvement in high-temperature stability, low-temperature crack resistance, and water stability of the mixtures. Recent studies also indicate that under heavy-load conditions, basalt fibers can significantly enhance the dynamic modulus, rutting resistance, and fatigue life of modified asphalt mixtures, providing more detailed experimental evidence for the application of fibers in engineering structures [20].

When fibers are added to high modulus asphalt concrete, the fibers combine with asphalt, aggregates, and high-modulus agent particles to form a three-dimensional network structure. This structure helps to bear and distribute stress [18,21]. Due to the oil-absorbing properties of the fibers, they increase the proportion of structural asphalt in the mixture, thereby improving the viscosity of the asphalt [22]. At the same time, the fibers restrict the movement of both asphalt and aggregates, which helps to hinder crack propagation. Moreno-Navarro et al. [23] conducted performance tests on high modulus asphalt concrete under high and low temperatures, water, and freezing conditions by adding acrylic fibers. They determined that the addition of fibers significantly improves the mechanical properties of high modulus asphalt concrete under harsh environmental conditions. Yan [24] prepared high modulus asphalt concrete using different methods and compared the performance changes in the mixture after adding hard asphalt, HMAM additives, and polyester fibers. The results showed that polyester fibers not only maintain the rutting resistance of high modulus asphalt concrete but also effectively improve the crack resistance of the mixture. Peressutti et al. [25] developed high modulus asphalt concrete with strong high-temperature performance and good low-temperature crack resistance by adding waste asphaltene and PET fibers. The performance was validated through dynamic modulus and tensile strength tests under high and low temperature conditions. Wang et al. [26] proposed the use of Trinidad Lake Asphalt (TLA) and polyester fibers to prepare high modulus asphalt concrete. Based on laboratory accelerated loading, low-temperature bending, freeze–thaw splitting, and APA fatigue tests, they studied the effects of TLA and polyester fiber content on the mixture’s high-temperature stability, water stability, low-temperature crack resistance, and fatigue resistance. The study also revealed the composite modification mechanism of TLA and polyester fibers.

In summary, fiber reinforcement technology provides a new approach for improving the overall performance of asphalt mixtures. Existing studies have shown that materials such as basalt fiber, polyester fiber, and lignin fiber can enhance the toughness of the mixture, improving its crack resistance and fatigue resistance [27]. However, existing studies on the regularity of fiber’s impact on the performance of high modulus asphalt concrete are relatively scarce. Furthermore, a scientific and systematic performance evaluation system for fiber-reinforced high modulus asphalt concrete has not yet been established, which somewhat restricts the further development and application of this technology.

In this study, basalt fibers (BF), polyester fibers (PF), and lignin fibers (LF) were selected to reinforce high-modulus asphalt mixtures. This is because BF have high strength, large modulus, and excellent heat resistance, which can improve high-temperature rutting resistance and low-temperature crack resistance; PF possess good toughness and elasticity, which are beneficial for fatigue resistance; LF, as natural fibers, can improve low-temperature performance and water stability, and are also cost-effective and environmentally friendly. Based on the aforementioned background, this study systematically investigates the high-temperature, low-temperature, fatigue, and water stability properties of fiber-reinforced high modulus asphalt concrete through various pavement performance tests, including rutting tests, low-temperature bending tests, immersion Marshall tests, freeze–thaw splitting tests, fatigue tests, and dynamic modulus tests. Through a comparative analysis of the test results, this study examines the impact of basalt fiber, polyester fiber, and lignin fiber on the performance characteristics of high modulus asphalt concrete. The recommended fiber contents for each type of fiber are determined, and a performance evaluation system for fiber-reinforced high modulus asphalt concrete is developed. This provides both theoretical foundation and technical support for the engineering applicability assessment and pavement structure optimization of fiber-reinforced high modulus asphalt concrete.

## 2. Materials and Methods

### 2.1. Materials

The asphalt selected for this study was 70# matrix asphalt supplied by Sinopec Qilu Petrochemical Company (Zibo, China). Both coarse and fine aggregates, as well as mineral filler, were derived from crushed limestone and provided by Shaanxi Jianning Construction Materials Co., Ltd. (Xi’an, China). The high-modulus agent used in this study was HXR, which was supplied by Jiangsu Zengguang New Materials Technology Co., Ltd. (Yancheng, China). The fibers employed included basalt fiber (BF), polyester fiber (PF), and lignin fiber (LF), all of which were supplied by Suzhou CelluTech Chemical Co., Ltd. (Suzhou, China). The performance indicators of various raw materials were tested in accordance with JTG E20-2011 [28], JTG 3432-2005 [29] and JT/T 533-2020 [30]. The test results are shown in Table 1, Table 2, Table 3 and Table 4.

### 2.2. Gradation Design

**Figure 1:**
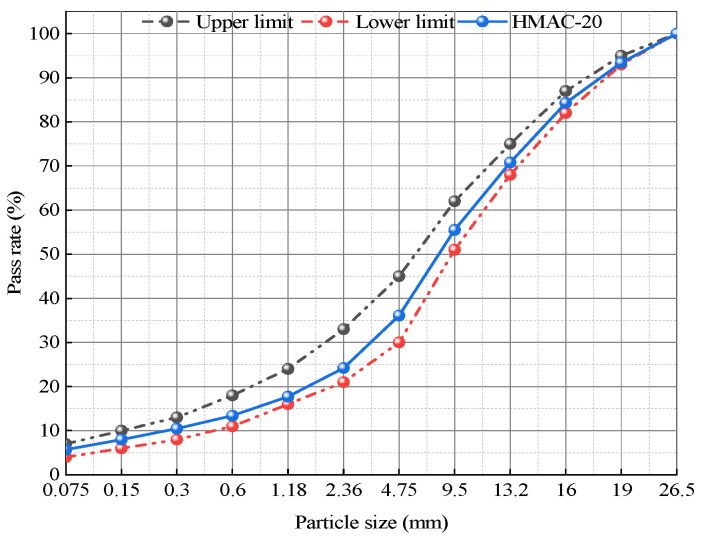
Gradation curve of HMAC-20.

Based on the previous research results of the project team, this study uses a medium-grade high modulus asphalt concrete with 20 mm nominal maximum aggregate size (HMAC-20) as the target gradation for HMAC [31,32]. Based on the flat S-shaped design principle, the proportions of coarse and fine aggregates were optimized to achieve a smooth S-shaped gradation curve for the mixture, forming a skeleton interlock structure that balances both the compactness and stability of the mixture. The gradation curve of HMAC-20 is shown in Figure 1.

Based on practical engineering and theoretical experience, the dosage of additives is determined as a proportion of the total mass of the asphalt mixture. The HXR-type high modulus agent content is set at 0.4%, with basalt fiber content at 0.3%, 0.4%, and 0.5%, and polyester fiber and lignin fiber content both at 0.2%, 0.3%, and 0.4%. The fibers are added individually to prepare fiber-reinforced high modulus asphalt concrete. The specific types of asphalt mixtures for each group are shown in Table 5.

### 2.3. Test Methods

#### 2.3.1. Rutting Test

This study uses the rutting test to evaluate the high-temperature deformation resistance of asphalt mixtures. First, plate-shaped specimens are prepared using a wheel rolling instrument. Since the high modulus agent is a polymer, polymer-modified asphalt mixtures require the specimens to be left together with the molds at room temperature for at least 48 h. After that, the asphalt mixture specimens are kept at the test temperature for conditioning. The rotating link is adjusted to reduce the contact pressure of the test wheel until a pressure of 0.7 MPa is applied to the surface of the asphalt mixture specimen. The test is concluded after 1 h. Dynamic stability primarily reflects the ability of the mixture to resist deformation under constant loading, whereas relative deformation represents the accumulated settlement or permanent deformation of the mixture over a certain testing duration or number of load cycles. By examining both indicators simultaneously, the high-temperature stability of the mixture can be evaluated more comprehensively and accurately. The dynamic stability (DS) is then calculated based on the rut deformation at 45 min and 1 h. The relative deformation parameter (PRD) is also used as an evaluation index. The formulas for calculating DS and PRD are shown in Equations (1) and (2).

(1) $$DS=\frac{N(t_{2}-t_{1})}{d_{2}-d_{1}}C_{1}C_{2}$$

In the equations, *DS* is the dynamic stability (cycles/mm); *N* is the number of wheel passes per minute (passes/min); *d*_1_ and *d*_2_ are the deformation values of the specimen at times *t*_1_ and *t*_2_ (mm); and *C*_1_ and *C*_2_ are experimental correction factors, both taken as 1.0 in this test.

(2) $$PRD=\frac{R_{60\min}}{D}\times 100\%$$

In the equation, *PRD* is the relative deformation (%); *R*_60min_ is the permanent deformation at the 60 min mark (mm); and *D* is the thickness of the rutting specimen, which is 50 mm in this test.

#### 2.3.2. Low-Temperature Bending Tests

As a commonly used method for evaluating the low-temperature performance of asphalt mixtures, the low-temperature bending beam test is simple to operate and widely applied. First, plate-shaped specimens are prepared and cut into prismatic beams. The prepared specimens are then placed in an environment at the required temperature for conditioning. Once the specimens reach the desired temperature, the test is conducted using a Universal Testing Machine (UTM). The beam supports are installed, the specimen is placed, and the span is set to 200 mm, with the upper platen positioned exactly at the center of the specimen. A concentrated load is applied at a rate of 50 mm/min until the specimen breaks under the load. During the test, the load-deflection curve is recorded in real-time. Using the program, the values of *R_B_*, *ε_B_*, and *S_B_* at the point of failure are calculated based on Equations (3)–(5).

(3) $$R_{B}=\frac{3LP_{B}}{2bh^{2}}$$

(4) $$\varepsilon_{B}=\frac{6hd}{L^{2}}$$

(5) $$S_{B}=\frac{R_{B}}{\varepsilon_{B}}$$

In the equation: *R_B_* is the flexural tensile strength (MPa); *P_B_* is the maximum load (N); *ε_B_* is the strain at failure (με); *L* is the span length (mm); *b* is the cross-sectional width (mm); *h* is the cross-sectional height (mm); *d* is the deflection at the section (mm); and *S_B_* is the stiffness modulus (MPa).

#### 2.3.3. Water Stability Test

This study uses the immersion Marshall test and freeze–thaw splitting test to investigate the mixture’s resistance to water damage. Both types of tests use standard Marshall specimens, which are randomly divided into four groups and placed in a 60 °C constant temperature water bath for curing. One group is placed for 30 min, while another group is placed for 48 h. The Marshall stability (*MS*_1_) and the stability after immersion (*MS*_2_) are measured, and the immersion residual stability of the asphalt mixture is calculated using Equation (6). In the other two groups, one is placed in a 25 °C water bath for 2 h, while the other is first vacuum-saturated and then conditioned for 16 h in an environment at −18 °C. After this, the specimen is placed in a 60 °C water bath for 24 h. The splitting load of each group of asphalt mixtures is measured, and the splitting tensile strength (*R_T_*_1_ and *R_T_*_2_) is calculated using Equation (7). The freeze–thaw splitting strength ratio is then calculated using Equation (8).

(6) $$MS_{0}=\frac{MS_{2}}{MS_{1}}\times 100\%$$

In the equation: *MS*_0_ is the immersion residual stability (%); *MS*_1_ is the standard stability (kN); and *MS*_2_ is the stability after immersion (kN).

(7) $$R_{T}=0.06287\times \frac{P_{T}}{h}$$

(8) $$TSR=\frac{R_{T2}}{R_{T1}}\times 100\%$$

In the equation: *R_T_* is the splitting tensile strength (MPa); *P_T_* is the maximum test load (N); h is the specimen height (mm); *R_T_*_2_ is the splitting tensile strength after freeze–thaw (MPa); *R_T_*_1_ is the splitting tensile strength (MPa); and *TSR* is the freeze–thaw splitting strength ratio (%).

#### 2.3.4. Four-Point Bending Fatigue Test

This study evaluates the fatigue performance of asphalt mixtures through a four-point bending fatigue test. A strain-controlled loading method is used. First, plate-shaped specimens of asphalt mixture, measuring 400 mm in length, 300 mm in width, and 75 mm in height, are prepared and then cut into prisms of 380 mm × 63.5 mm × 50 mm. These specimens are placed in a 15 ± 0.5 °C environmental chamber and cured for no less than 4 h. Three strain control levels of 400 με, 500 με, and 600 με are selected. A 10 Hz loading mode is used, and prior to the formal test, 50 loading cycles at the specified strain levels are applied to obtain the initial stiffness modulus of the mixture. The formal test begins once the strain level stabilizes at the specified level. The test is automatically terminated when the specimen’s stiffness modulus decreases to 50% of its initial modulus. The number of loading cycles at this point is recorded as the fatigue life.

#### 2.3.5. Dynamic Modulus Test

The dynamic modulus of each group of asphalt mixtures is tested using the Asphalt Mixture Performance Tester. First, the initial stress and strain thresholds are preset to preload the specimen. During this stage, data is collected to achieve system self-calibration. Once the system stabilizes, dynamic loading is applied at the set frequency, and data is collected to calculate the material’s dynamic compressive modulus. Before the test, cylindrical specimens with a diameter of 150 mm and a height of 170 mm are prepared using a gyratory compactor. These are then cored and cut to the required size of φ100 mm × 150 mm using a drilling machine and a cutting machine. It is important to strictly control the temperature and compaction cycles during specimen preparation. Otherwise, if the void content of the specimen does not meet the test requirements, the accuracy of the measured dynamic modulus cannot be ensured. Before the test, the specimens are placed in an environmental chamber for 5 h of temperature equilibrium under three temperature gradients (20 °C, 35 °C, 50 °C). During the test, an axial sinusoidal load is applied to the specimens at five loading frequencies (25 Hz, 20 Hz, 10 Hz, 1 Hz, and 0.1 Hz). The deformation data of the specimens is monitored in real-time using three-dimensional displacement sensors, and the system automatically calculates the dynamic modulus and phase angle of the asphalt mixture at each corresponding frequency.

## 3. Results

### 3.1. High-Temperature Performance

**Figure 2:**
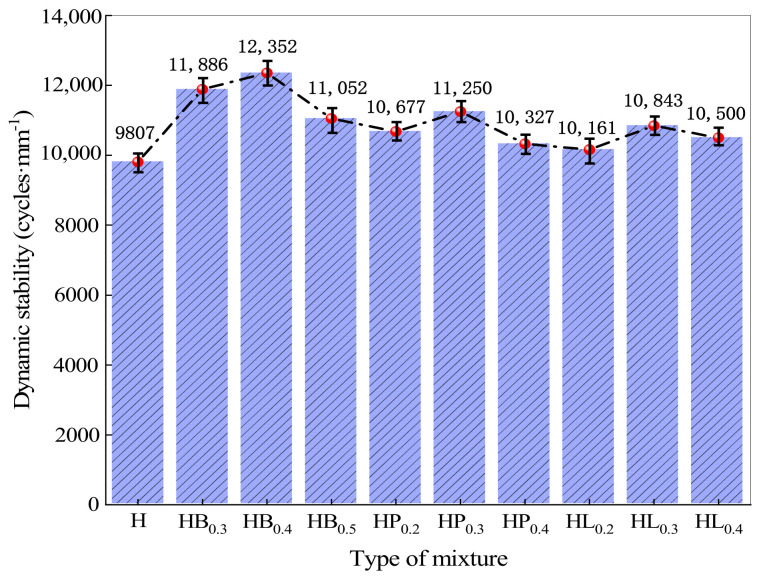
Rutting test results.

The rutting test results for each group of asphalt mixtures, including dynamic stability and relative deformation, are shown in Table 6 and Figure 2.

The rutting test results of each asphalt mixture, including dynamic stability and relative deformation, are presented in Table 6 and Figure 2. The results indicate that after the addition of basalt fiber, polyester fiber, or lignin fiber alone, the dynamic stability of the mixtures first increases and then decreases with increasing fiber content, while the relative deformation first decreases and then increases. When the fiber contents are 0.4%, 0.3%, and 0.3% for the three fibers, respectively, the dynamic stability of the mixtures is 1.26, 1.14, and 1.11 times that of the high-modulus asphalt mixture. Analysis of the relative deformation of the asphalt mixtures shows that its variation trend is consistent with that of dynamic stability, both exhibiting an initial increase followed by a decrease with increasing fiber content.

The addition of fibers improves the high-temperature performance of high modulus asphalt concrete. This enhancement is attributed to the interactions among fibers, asphalt, the high-modulus agent, and aggregates, which contribute to internal load transfer and stress distribution within the mixture. Fibers have a large specific surface area, allowing them to adsorb free asphalt and increase the proportion of structural asphalt [33]. In this study, the improved high-temperature stability observed in rutting tests supports the effect of fiber incorporation on resistance to permanent deformation under applied loads [34]. This enhances the contact between structural asphalt and aggregates, thereby improving the cohesion between them and enhancing the high-temperature deformation resistance of the asphalt mixture.

The reinforcing effect of basalt fiber on high modulus asphalt concrete is more pronounced than that of polyester fiber and lignin fiber. Polyester fiber and lignin fiber, due to their relatively large specific surface areas, can adsorb a considerable amount of asphalt, which increases the content of structural asphalt and enhances aggregate interlock, thereby improving the overall integrity of the mixture. Basalt fiber, however, has a higher modulus and can form a more stable three-dimensional network within the mixture [35]. This enables it to better transfer and distribute thermal stress, resulting in greater resistance to high-temperature deformation. The differences in performance among the fibers are consistent with the high-temperature rutting test results presented in this study.

However, as shown in Figure 2, with a further increase in fiber content, the dynamic stability of fiber-reinforced high modulus asphalt concrete does not improve but rather decreases, dropping by 10.5%, 7.7%, and 6.3% compared to the previous fiber content levels. At the same time, rut depth and relative deformation increase. When the fiber content is excessively high, fibers cannot be uniformly dispersed in the HMAC and tend to agglomerate, forming localized fiber-rich regions that disrupt the microstructural continuity of the asphalt–aggregate–fiber composite system. Fiber agglomeration weakens the effective asphalt coating on aggregates and introduces interfacial defects, thereby reducing the bonding performance between asphalt and aggregates. Under high-temperature loading, these agglomerated regions are unable to effectively participate in load bearing; instead, they interrupt stress transfer pathways and induce stress concentration, resulting in structural weak zones. Consequently, the deterioration of microstructural integrity and non-uniform stress transfer jointly account for the decline in high-temperature stability of the high-modulus asphalt mixture at excessive fiber contents.

### 3.2. Low-Temperature Crack Resistance

**Figure 3:**
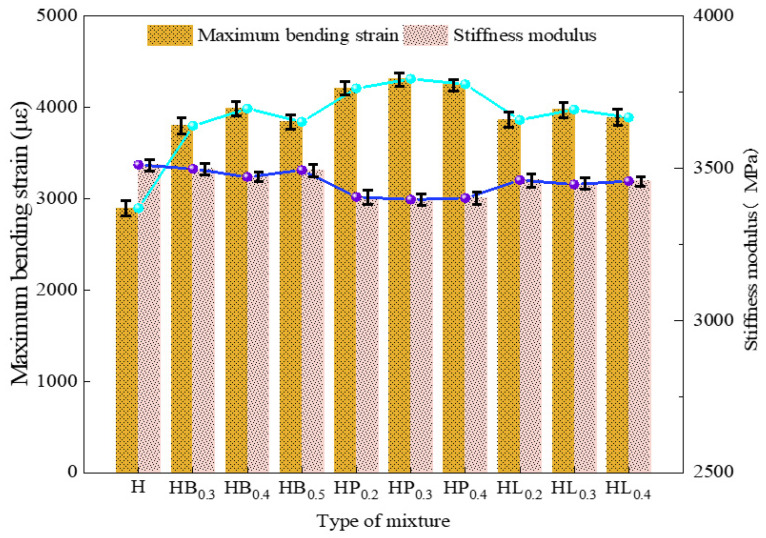
Low-temperature performance test results.

The bending tensile strength, maximum bending strain, and stiffness modulus of each group of asphalt mixtures are presented in Table 7 and Figure 3.

Regardless of the type of fiber added, the bending strength and flexural tensile strain of the mixture increased, while the stiffness modulus decreased. It can be observed that the incorporation of fibers significantly improves the low-temperature performance of high modulus asphalt mixtures. Moreover, as the fiber content increases, the enhancement effect first intensifies and then diminishes. When fibers were incorporated individually at contents of 0.4% basalt fiber, 0.3% polyester fiber, and 0.3% lignin fiber, the asphalt mixtures exhibited the best low-temperature performance, with the failure strain increased by 37.6%, 48.9%, and 37.2%, respectively. During the initiation and propagation of cracks, stress concentration develops at the crack tips within the mixture. Because the fibers possess physical properties—such as elastic modulus, tensile strength, and flexibility—that differ markedly from those of the asphalt mixture, residual stress–strain fields and microcracks are generated at the fiber–mixture interfaces. These effects help to reduce crack-tip stress concentration and enhance the flexibility of the asphalt mixture. In addition, fibers can inhibit crack initiation and propagation through reinforcement and bridging effects, thereby improving the low-temperature cracking resistance of the mixture.

With a high level of fiber addition, the enhanced asphalt absorption by fibers reduces the effective asphalt content in the mastic and induces local stiffening, thereby weakening the flexibility and strain accommodation capacity of the mixture under low-temperature conditions. At low temperatures, the asphalt binder inherently becomes more brittle, and the stiffness mismatch between fiber-enriched regions and the surrounding matrix is further amplified, making uniform strain release difficult and promoting stress concentration at the interfaces. Unlike an appropriate fiber content, which can bridge microcracks and delay crack propagation, excessive fibers contribute little to crack energy dissipation or crack blunting. As a result, cracks are more likely to initiate and propagate rapidly under thermal shrinkage stress or external loading, leading to a reduction in the low-temperature cracking resistance of the high-modulus asphalt mixture.

The addition of 0.3% polyester fiber provides the most pronounced improvement in the low-temperature cracking resistance of high modulus asphalt mixtures. Compared with the high modulus asphalt mixture, the failure strain increases by 48.9% while the stiffness modulus decreases by 11.3%. Basalt fiber shows the second-best enhancement effect, whereas lignin fiber exhibits the least improvement. The underlying mechanism can be explained as follows: owing to its high tensile strength, basalt fiber forms a three-dimensional network structure within the mixture, which effectively suppresses the initiation and propagation of cracks; polyester fiber, on the other hand, has stronger asphalt absorption, a density close to that of asphalt, and better compatibility with asphalt. It can adsorb a large amount of asphalt, increasing the proportion of structural asphalt. Moreover, polyester fiber has a higher fracture elongation and greater flexibility, enhancing the mixture’s ability to resist cracking under external loads, and thus provides a greater improvement in the low-temperature performance of the mixture; lignin fiber has lower flexibility than polyester fiber and lower strength than basalt fiber; therefore, its improvement effect is intermediate between the two.

### 3.3. Water Stability

**Figure 4:**
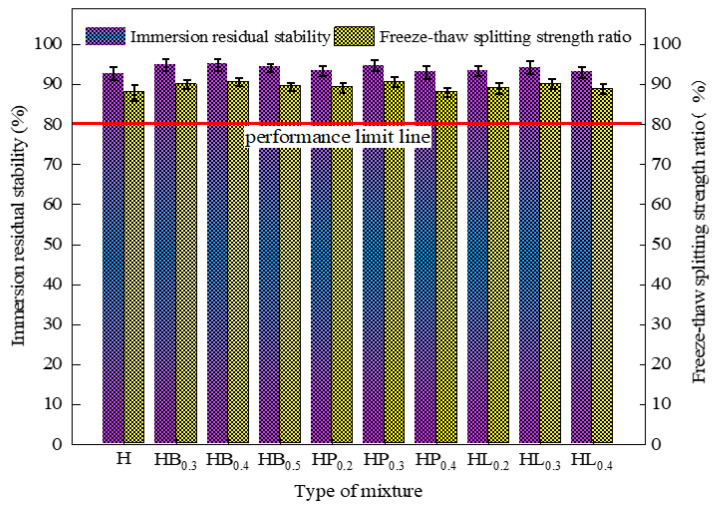
Water stability test results.

The test indicators, including residual stability after immersion and splitting strength ratio of each asphalt mixture, are presented in Table 8 and Figure 4. It can be observed that both the residual stability of high-modulus asphalt mixtures with and without fibers exceeds 90%, and the splitting strength ratio exceeds 85%, all of which meet the requirements of the JTG F40 specification.

The test data indicate that the addition of fibers slightly improves the water stability of high modulus asphalt mixtures, but the magnitude of change is minimal. The effectiveness of fibers in improving water stability follows the order: basalt fiber > polyester fiber > lignin fiber. Fibers can adsorb free asphalt, increasing its viscosity, and their reinforcement and anchoring effects help delay water infiltration. Therefore, they have a certain positive effect on water stability. However, since high modulus asphalt mixtures already possess excellent inherent water stability, the enhancing effect of fibers is not pronounced. As the fiber dosage exceeds the optimal level, the enhanced asphalt absorption by fibers weakens the effective coating of aggregates by the asphalt mastic, thereby reducing the adhesion stability at the asphalt–aggregate interface. Meanwhile, the introduction of excessive fibers alters the internal pore structure of the mixture and increases pore connectivity, providing pathways for water migration and retention within the mixture. Under moisture intrusion, competitive adsorption of water occurs at the asphalt–aggregate interface, leading to interfacial debonding and stripping damage. The progressive accumulation of interfacial damage under coupled moisture–mechanical actions ultimately results in a decline in the water stability of the high-modulus asphalt mixture with increasing fiber content.

### 3.4. Fatigue Resistance

**Figure 5:**
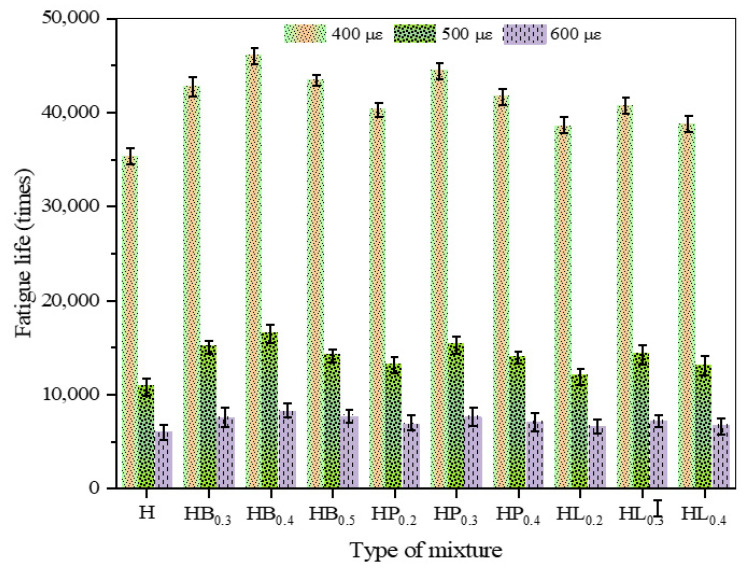
Four-point bending fatigue test results.

As shown in Table 9 and Figure 5, with the increase in strain level, the fatigue life of all asphalt mixtures exhibits a significant decreasing trend. At the same strain level, the addition of fibers increases the fatigue life of high-modulus asphalt mixtures, and the fatigue life first increases and then decreases with increasing fiber content. Specifically, when the contents of basalt fiber, polyester fiber, and lignin fiber are 0.4%, 0.3%, and 0.3%, respectively, the fatigue life at a strain level of 400 με is increased by 30.5%, 26.1%, and 15.3%.

At 15 °C, asphalt mixtures exhibit good viscoelastic behavior under external loading, with bending or tensile deformations ultimately developing into flexible fracture. A higher stiffness modulus allows the mixture to recover more deformation during each strain cycle, enabling it to withstand more repeated loads before fatigue failure, thereby enhancing its fatigue performance. The high modulus agent can combine with asphalt components to form a strong and stable polymer chain structure, increasing the adhesion between asphalt and aggregates and improving the mechanical properties of the mixture. This significantly enhances the stiffness modulus of the asphalt mixture, thereby improving the fatigue resistance of high modulus asphalt mixtures.

After adding fibers, when the asphalt mixture is subjected to bending under external loads, the fibers—due to their reinforcement, anchoring effects, strength, and flexibility—allow the mixture to withstand more bending cycles, effectively enhancing the fatigue resistance of the high modulus asphalt mixture. Moreover, as the fiber content increases, the fatigue resistance of the mixture improves. At excessively high fiber contents, the internal fiber network within the mixture fails to form an effective continuous reinforcement structure, limiting the fibers’ ability to bridge cracks and dissipate energy under cyclic loading. At the microscale, fibers are unable to fully participate in stress transfer and distribution, causing local strain concentration regions to accumulate damage more easily and accelerating the initiation and propagation of microcracks. With increasing cyclic loads, these microcracks progressively connect and extend, leading to a reduction in the overall fatigue performance of the mixture. Excessive fibers not only fail to further improve fatigue life, but also diminish the material’s capacity to resist repeated stress. Basalt fiber has a high elastic modulus and strength, exhibiting excellent mechanical properties; therefore, it provides the greatest improvement in the resistance of high modulus asphalt mixtures to fatigue failure. Polyester fiber has greater flexibility and fracture elongation, lower modulus and strength, so its improvement effect is not as pronounced as that of basalt fiber. Lignin fiber has lower strength than basalt fiber and less flexibility than polyester fiber, resulting in the least improvement effect.

### 3.5. Dynamic Modulus Test

**Figure 6:**
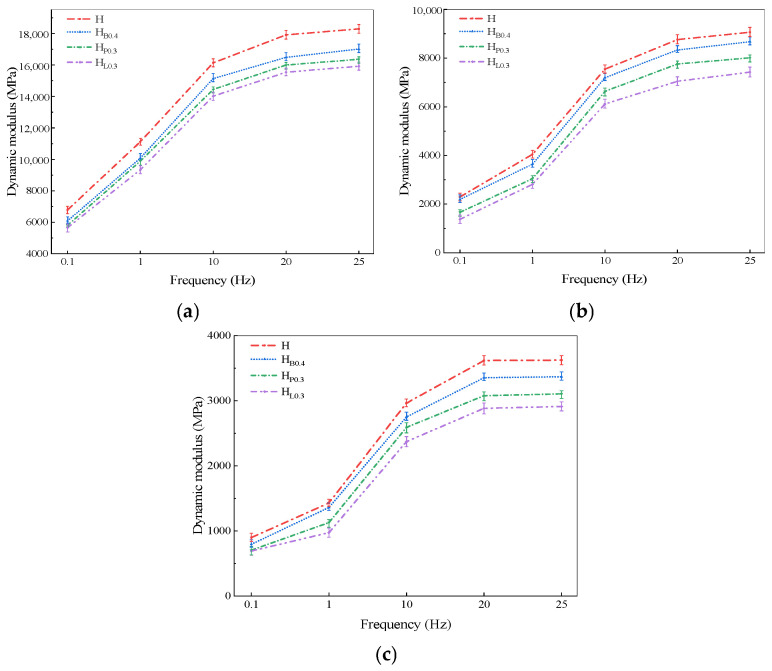
Dynamic modulus–frequency relationship curve: (**a**) 20 °C; (**b**) 35 °C; (**c**) 50 °C.

The dynamic modulus test measured the dynamic modulus of the matrix asphalt mixture, high modulus asphalt mixture, and fiber-reinforced high modulus asphalt mixture with the optimal fiber content at temperatures of 20 °C, 35 °C, and 50 °C, and at loading frequencies of 25 Hz, 20 Hz, 10 Hz, 1 Hz, and 0.1 Hz. The results are presented in Table 10 and Figure 6.

The test results show that the dynamic modulus of all five asphalt mixtures follows a similar trend with changes in temperature and frequency. As the test temperature rises, the mechanical behavior of the asphalt mixture gradually transitions from viscoelastic to viscous, weakening the asphalt’s adhesive properties. Consequently, the mixture’s overall structural integrity and stability decrease, leading to a gradual reduction in its dynamic modulus under applied loads. At a constant temperature, as the loading frequency decreases, the dynamic modulus of the asphalt mixture gradually declines. This is due to the viscoelastic nature of the mixture, which causes a longer response time and larger strain in the specimen at lower frequencies, resulting in a reduced dynamic modulus. Under different loading frequencies, asphalt mixtures exhibit distinct mechanical behaviors: at lower frequencies, the mixture behaves more like a viscous material, while at higher frequencies, it approaches elastic behavior. As a result, the dynamic modulus of the asphalt mixture decreases as the frequency decreases.

A temperature of 20 °C is close to the annual average pavement temperature or the typical service temperature range in most regions of China. It falls within the moderate temperature range, reflecting the material’s stiffness under normal conditions without the modulus being excessively reduced by high temperatures. A frequency of 10 Hz corresponds to a vehicle speed of approximately 72–80 km/h, which aligns with the typical design speeds of vehicles on China’s high-grade highways. Therefore, taking the dynamic modulus data at 20 °C and 10 Hz as an example, the dynamic modulus of high modulus asphalt concrete increased by 54.6% compared with that of the base asphalt mixture. The high modulus agent forms polymeric chains within the asphalt, providing reinforcement and bonding, which restricts the movement of asphalt and aggregates and enhances the dynamic modulus of the asphalt mixture. After adding a single type of fiber, the dynamic modulus of high modulus asphalt concrete is lower than that of the mixture before fiber addition, as shown in Figure 6. It can be seen that after adding basalt fiber, polyester fiber, and lignin fiber, the dynamic modulus of high modulus asphalt concrete decreased by 7.04%, 9.31%, and 12.96%, respectively, compared with the mixture before fiber addition. The reason for this phenomenon is that fibers increase the air voids of high modulus asphalt concrete and, due to their flexibility and oil-absorbing properties, enhance the mixture’s flexibility, which reduces its stiffness and modulus, resulting in a decrease in dynamic modulus.

With the increase in fiber content, the dynamic modulus of HMAC exhibits a decreasing trend, whereas high-temperature stability and fatigue performance show a non-monotonic variation characterized by an initial increase followed by a decrease. Although the reduction in dynamic modulus reflects a decrease in the overall stiffness of the mixture, an appropriate amount of fibers can form a three-dimensional reinforcement network within the asphalt matrix, providing local support to the aggregate skeleton and improving stress distribution, thereby enhancing the mixture’s rutting resistance at high temperature and fatigue durability under cyclic loading. In other words, the dynamic modulus primarily reflects the overall viscoelastic characteristics of the mixture, while high-temperature stability and fatigue performance depend more on the micro-scale reinforcement network of fiber–aggregate–asphalt and its stress transfer mechanism. When the fiber content exceeds the optimal range, the fiber network is locally constrained or disrupted, weakening the reinforcement effect, and high-temperature stability and fatigue performance decrease accordingly. Therefore, a decrease in dynamic modulus does not necessarily lead to a decline in macroscopic performance, and both properties can exhibit an initial increase followed by a decrease within a certain range of fiber content, reflecting the regulatory role of fibers in microstructure and stress transfer.

Currently, international standards for the dynamic modulus of high modulus asphalt concrete mainly refer to the French technical specifications, which use the complex modulus at 15 °C and 10 Hz as the evaluation criterion, requiring that the dynamic modulus of high modulus asphalt concrete under these conditions should not be less than 14,000 MPa. According to JTG D50-2017 [36], the AC-20 graded asphalt concrete with 70# asphalt has a dynamic modulus range of 9000–13,500 MPa at 20 °C and 10 Hz. The recommended standard JT/T 860.8-2023 [37] specifies that high modulus asphalt concrete should have a dynamic modulus of no less than 13,000 MPa under the same conditions. Based on relevant research findings and a comprehensive assessment, it is recommended that the dynamic modulus of fiber-reinforced high modulus asphalt concrete at 20 °C and 10 Hz should be 13,000~18,000 MPa.

## 4. Conclusions

Based on the determination of the mixture gradation, this study introduces basalt fiber, polyester fiber, and lignin fiber to prepare three types of fiber-reinforced high modulus asphalt concrete. Rutting tests, low-temperature bending tests, immersion Marshall tests, freeze–thaw splitting tests, four-point bending fatigue test, and dynamic modulus tests were conducted to evaluate the improvement and impact of fibers on the performance of the mixture. The main conclusions are as follows:

(1) When fibers are added alone, the high-temperature rutting resistance of HMAC first increases and then decreases with increasing fiber content. Among them, the HMAC incorporating basalt fiber exhibits the best high-temperature performance, with the dynamic stability increased by 26% compared with the specimen without fiber addition.
(2) Fibers can improve the low-temperature performance of HMAC. The addition of polyester fiber provides the most significant enhancement in low-temperature cracking resistance, with the failure strain increased by 48.9% and the stiffness modulus reduced by 11.3%.
(3) Fibers can enhance the water stability of HMAC; however, the improvement is not pronounced. The effectiveness of fibers in improving water stability follows the order basalt fiber > polyester fiber > lignin fiber.
(4) After fiber addition, the fatigue life of HMAC is improved and exhibits a trend of first increasing and then decreasing. At the optimal dosages of the three fibers, the fatigue life is increased by 30.5%, 26.1%, and 15.3%, respectively, with basalt fiber providing the most significant improvement.
(5) The addition of fibers has a negative effect on the dynamic modulus. Under the conditions of 20 °C and 10 Hz, basalt fiber, polyester fiber, and lignin fiber reduce the dynamic modulus of HMAC by 6.24%, 6.92%, and 13.13%, respectively, with basalt fiber having a relatively smaller effect.
(6) Based on the evaluation of the pavement performance of HMAC reinforced with three types of fibers, the recommended dosages of basalt fiber, polyester fiber, and lignin fiber are 0.4%, 0.3%, and 0.3%, respectively.

## Figures and Tables

**Table 1:** Technical properties of 70# base asphalt.

Test Properties		Test Results	Requirements
Penetration (25 °C, 0.1 mm)		69	60~80
Penetration index		−1.0	−1.5~+1.0
Softening point (°C)		47.3	≥46
Dynamic viscosity (60 °C)		264	≥180
Ductility (10 °C, cm)		53	≥20
Ductility (15 °C, cm)		>150	≥100
Wax content (%)		1.8	≤2.2
Flash point (°C)		>300	≥260
Solubility (%)		99.96	≥99.5
Density (15 °C, g·cm^−3^)		1.038	Measured Records
After TFOT	Mass change (%)	−0.06	±0.8
	Penetration ratio (%)	67.6	≥61
	Ductility (10 °C, cm)	8	≥6

**Table 2:** Technical properties of aggregates.

Aggregate Types	Test Properties	Test Results	Requirements
Coarse aggregates	Aggregate crushing value (%)	10	≤28
	Soundness (%)	7	≤12
	Water absorption (%)	0.4	≤3
	Apparent specific gravity (g·cm^−3^)	2.768	≥2.5
	Flat and elongated particle content (%)	2	≤15
Fine aggregates	Apparent specific gravity (g·cm^−3^)	2.690	≥2.5
	Mud content (%)	0.9	≤3
Limestone powder	Apparent specific gravity (g·cm^−3^)	2.710	≥2.5
	Water content (%)	0.2	≤1

**Table 3:** Technical properties of HXR high modulus agent.

Test Properties	Test Results
Appearance	Granular, uniform, free of any binder.
Sieve Size (mm)	≤5
The mass of a single particle (g)	0.01~0.025
Density (g·cm^−3^)	0.84
Melt flow index (g·cm^−3^)	≥1.0
Ash content (%)	≤3.0

**Table 4:** Technical properties of fibers.

Test Properties	Basalt Fiber	Polyester Fiber	Lignin Fiber
Appearance	Golden-brown, regular, bundle-like clusters	White, filamentous strands	Gray, flocculent
Diameter (μm)	17	13.4	43
Length (mm)	6	6	4
Molecular weight	-	-	8500
Impurity content (%)	≤1	≤1	≤1
Moisture content (%)	0.1–0.5%	0.2–0.5%	3–5%
Density (g·cm^−3^)	2.80	1.36	1.25
Tensile strength (Mpa)	3500~4000	586	250
Elastic modulus (Gpa)	76.1	13.5	9
Melting point (°C)	1600	259	257
Fracture elongation (%)	2.99	21.0	-

**Table 5:** Type of asphalt mixture.

Mixture Types	Additives	High Modulus Agent Content (%)	Fiber Content (%)
H	HXR	0.4	0
H_B0.3_	HXR + BF	0.4	0.3
H_B0.4_	HXR + BF	0.4	0.4
H_B0.5_	HXR + BF	0.4	0.5
H_P0.2_	HXR + PF	0.4	0.2
H_P0.3_	HXR + PF	0.4	0.3
H_P0.4_	HXR + PF	0.4	0.4
H_L0.2_	HXR + LF	0.4	0.2
H_L0.3_	HXR + LF	0.4	0.3
H_L0.4_	HXR + LF	0.4	0.4

**Table 6:** Rutting test results.

Mixture Types	45 min Deformation (mm)	60 min Deformation (mm)	Dynamic Stability (Cycles/mm)	Relative Deformation (%)
H	1.540	1.605	9807	3.21
H_B0.3_	1.405	1.458	11,886	2.92
H_B0.4_	1.369	1.420	12,352	2.84
H_B0.5_	1.388	1.445	11,052	2.89
H_P0.2_	1.427	1.486	10,677	2.97
H_P0.3_	1.409	1.465	11,250	2.93
H_P0.4_	1.452	1.513	10,327	3.03
H_L0.2_	1.433	1.495	10,161	2.99
H_L0.3_	1.413	1.471	10,843	2.94
H_L0.4_	1.426	1.486	10,500	2.97

**Table 7:** Low-temperature performance test results.

Mixture Types	Bending Tensile Strength (MPa)	Maximum Bending Strain (με)	Stiffness Modulus (MPa)
H	9.77	2897	3372
H_B0.3_	12.65	3799	3329
H_B0.4_	12.85	3987	3239
H_B0.5_	12.74	3844	3314
H_P0.2_	12.72	4209	3021
H_P0.3_	12.91	4314	2992
H_P0.4_	12.80	4255	3007
H_L0.2_	12.39	3865	3205
H_L0.3_	12.56	3977	3158
H_L0.4_	12.43	3891	3194

**Table 8:** Water stability test results.

Mixture Types	Immersion Marshall Test			Freeze–Thaw Splitting Test		
	Marshall Stability (kN)	Stability After Immersion (kN)	Immersion Residual Stability (%)	Splitting Tensile Strength (kN)	Splitting Tensile Strength After Freeze–Thaw (kN)	Freeze–Thaw Splitting Strength Ratio (%)
H	13.41	12.43	92.71	1.26	1.11	87.93
H_B0.3_	14.06	13.48	94.88	1.31	1.17	89.92
H_B0.4_	14.22	13.67	95.08	1.35	1.22	90.61
H_B0.5_	14.17	13.38	94.32	1.28	1.15	89.47
H_P0.2_	13.94	13.16	93.47	1.28	1.14	89.36
H_P0.3_	14.05	13.44	94.56	1.32	1.19	90.57
H_P0.4_	14.03	13.20	93.13	1.28	1.13	88.08
H_L0.2_	13.72	12.92	93.35	1.27	1.13	89.15
H_L0.3_	13.98	13.29	94.12	1.30	1.17	90.13
H_L0.4_	13.81	12.99	93.20	1.28	1.14	88.87

**Table 9:** Four-point bending fatigue test results.

Mixture Types	400 με		500 με		600 με	
	Stiffness Modulus (MPa)	Fatigue Life (Cycles)	Stiffness Modulus (MPa)	Fatigue Life (Cycles)	Stiffness Modulus (MPa)	Fatigue Life (Cycles)
H	9020	35,299	9166	10,899	8950	6039
H_B0.3_	9737	42,763	9587	15,057	9212	7535
H_B0.4_	9883	46,087	9592	16,454	9289	8195
H_B0.5_	9801	43,407	9590	14,166	9222	7711
H_P0.2_	9641	40,383	9455	13,143	9101	6875
H_P0.3_	9698	44,465	9508	15,299	9131	7612
H_P0.4_	9623	41,717	9472	13,968	9114	7084
H_L0.2_	9511	38,531	9345	11,955	9108	6578
H_L0.3_	9599	40,693	9408	14,342	9115	7183
H_L0.4_	9524	38,761	9386	13,011	9102	6699

**Table 10:** Dynamic modulus test results.

Mixture Types	Temperature (°C)	Frequency				
		25 Hz	20 Hz	10 Hz	1 Hz	0.1 Hz
H	20	18,293	17,914	16,136	11,121	6785
	35	9063	8758	7544	4043	2292
	50	3624	3619	2965	1430	899
H_B0.4_	20	17,010	16,479	15,129	10,079	6093
	35	8674	8335	7196	3638	2193
	50	3370	3355	2751	1362	795
H_P0.3_	20	16,359	15,996	14,440	9891	5807
	35	8012	7755	6632	3043	1664
	50	3107	3079	2592	1128	704
H_L0.3_	20	15,919	15,539	14,016	9337	5663
	35	7421	7047	6111	2809	1374
	50	2912	2884	2374	973	692

## Data Availability

The testing and analysis data used to support the findings of this study are included within the article.

## Abbreviations

The following abbreviations are used in this manuscript:

HMAC	High modulus asphalt concrete
BF	Basalt fiber
PF	Polyester fiber
LF	Lignin fiber
